# Mean Canal-body Ratio among Specimens of Dried Lumbar Vertebrae in the Department of Anatomy of a Medical College: A Descriptive Cross-sectional Study

**DOI:** 10.31729/jnma.7328

**Published:** 2022-04-30

**Authors:** Iju Shrestha

**Affiliations:** 1 Department of Anatomy, Kathmandu Medical College and Teaching Hospital, Duwakot, Bhaktapur, Nepal

**Keywords:** *anatomy*, *bones*, *lumbar vertebrae*

## Abstract

**Introduction:**

Lumbar spinal canal stenosis is assumed to be one of the chief causative factors for low back pain. The measurement of lumbar canal and body dimensions has thus become an important tool for the diagnosis and treatment of spinal stenosis. This study aims to find out the mean canal-body ratio among specimens of dried lumbar vertebrae in a medical college.

**Methods:**

A descriptive cross-sectional study was done in a medical college from May, 2021 to July, 2021. Ethical clearance was taken from the Institutional Review Committee (Reference number: 0502202103) and whole sampling was done. Seventy-three intact dried lumbar vertebrae were studied for the dimensions of the body and canal in transverse and anteroposterior planes. The findings were recorded and the canal body ratio was calculated using the transverse diameters of the spinal canal and vertebral body. The data obtained were computed and analysed using Microsoft Excel 2013. Point estimate at 95% Confidence Interval was calculated along with mean and standard deviation for continuous data.

**Results:**

The mean canal-body ratio was observed to be 0.53±0.032. The vertebral canal-body ratio was observed to be 0.58 in L1 followed by 0.53 in L2, 0.51 in L3, 0.49 in L4 and 0.53 in L5.

**Conclusions:**

The mean canal-body ratio observed in the present study was comparable to studies done in similar settings.

## INTRODUCTION

Back pain, especially in the lower back is seen as one of the common health problems. Various causes have been attributed to low backache, of which lumbar spinal canal stenosis is of great interest.^1^ Since narrowing of the spinal canal was believed to be the chief causative factor of stenosis, the dimensions of the spinal canal were studied previously.

Recently, it has been pointed out that it would be more reliable if the ratio of the vertebral canal and of vertebral body i.e. canal-body ratio is taken as an index for calculating the degree of stenosis.^2^ The measurement of dimensions of the lumbar canal and body has thus become an important tool for the diagnosis and treatment of spinal stenosis.^3-5^

The present study aims to find out the mean canal-body ratio among specimens of dried lumbar vertebrae in a medical college.

## METHODS

This was a descriptive cross-sectional study that was carried out in a medical college from May, 2021 to July, 2021. The study included all the lumbar vertebrae available in the Department of Anatomy, Kathmandu Medical College and Teaching Hospital. Ethical clearance from the Institutional Review Committee (Reference number: 0502202103) was obtained. The whole sampling was done.

The sample size was calculated using the formula,

n = Z^2^ × σ / e^2^

  = 1.96^2^ × 0.04^2^ / 0.01^2^

  = 62

Where,

n = minimum required sample size
Z = 1.96 at 95% Confidence Interval (CI)
σ = standard deviation taken as 0.04 (educated guess)
e = margin of error, 0.01

The minimum required sample size was 62. However, a sample size of 73 was taken including all the dried lumbar vertebrae were from the unidentified adult bodies available in the department for the study purpose. Transverse diameters of the spinal canal and the vertebral body were respectively recorded followed by the canal body ratio. All the dry lumbar bones with intact morphology were included in the study whereas the bones with damaged/broken morphology were excluded. For the measurements of the bone, the transverse diameter of the spinal canal was obtained by measuring the minimum distance between the pedicles of the same vertebra; whereas the diameter of the body was taken as the mid vertebral distance between the points on lateral borders of the vertebral body. The measurements were carried out with the help of a vernier calliper. The measurements were used to calculate the canal-body (C/B) ratio as follows: C/B = Transverse diameter of spinal canal/Transverse diameter of vertebral body.^1^

The data obtained were computed and analysed using Microsoft Excel 2013 to tabulate the results. Point estimate at 95% Confidence Interval was calculated along with mean and standard deviation for continuous data.

## RESULTS

**Figure 1:**
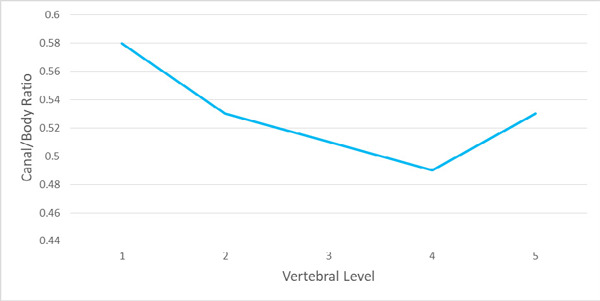
Canal-body ratio at different vertebral levels.

A total of 73 dry lumbar vertebrae were studied for their body and canal diameters, which further were used to tabulate the canal-body ratio. The vertebral canal-body ratio was observed to be 0.58 in L1 followed by 0.53 in L2, 0.51 in L3, 0.49 in L4 and 0.53 in L5 (Figure 1).

**Figure 2:**
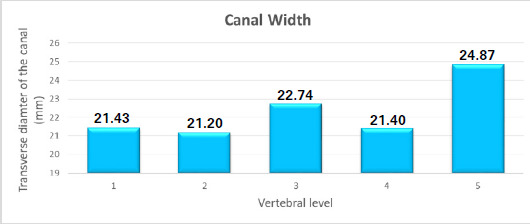
Transverse diameter of the canals at different levels.

The transverse diameter of the canal was also observed to be 21.4 mm in L1 and L4 whereas 21.2 mm and 22.74 mm in L2 and L3. It was maximum (24.87 mm) in L5 level. The mean was seen to be 22.33±1.78 (Figure 2).

**Figure 3:**
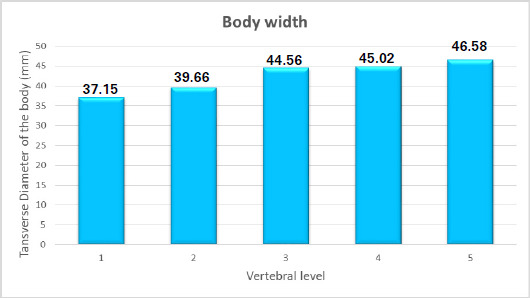
Transverse diameter of the body at different levels.

The transverse body width of the vertebrae showed a gradual increase in diameter. The minimum was observed in L1 and the maximum at L5 level with a mean of 42.59±3.57 (Figure 3).

**Figure 4:**
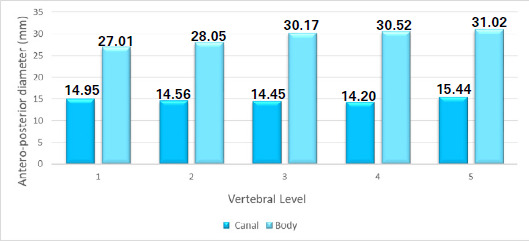
Antero-posterior diameters of the canal and body at different vertebral levels.

The anteroposterior diameter (AP) of the canal was observed to be almost constant till L4 with a slight increase at L5 with a mean of 14.72±0.43. Meanwhile, the mean AP dimension of the body was calculated to be 29.35±1.55 (Figure 4).

## DISCUSSION

Among different factors thought to be associated with low back pain, one is a change in diameter of the vertebral canal due to any pathological cause. Lumbar spinal canal stenosis has been looked into with great interest for being one of the causative factors for low back pain. For its diagnosis and treatment, the assessment of dimensions of the lumbar canal and body has been considered one of the important tools.^6^

In this study, the transverse diameter of the canal showed an increase at the level of L3, following a decrease in L2 but again showed a drop at L4 followed by an increase in diameter at the L5 level. Few studies have reported a gradual increase in the transverse diameter of the vertebral canal from L1 to L5, minimum being at L1 and maximum at L5.^7-10^ However, the finding of the present study is contrary to the finding of a study which has reported a decreasing transverse diameter of the lumbar vertebral canal from L1 to L5.^11^ On the other hand, a study found that the transverse diameter was constant at L1 and L2 and then increased gradually till L5 vertebra.^12^ This variation may be attributed to and as an adaptation to accommodate sacral nerve roots during angular movements of the vertebral column.

The transverse body width of the vertebrae in this study showed a gradual increase in diameter; the minimum was observed in L1 and the maximum at the L5 level with a mean of 42.59. The finding is consistent with that of the study done in the central and western regions of Nepal.^7,8^ Same pattern in the lower three lumbar vertebrae was observed by a study.^13^ Cranio-caudal increase in transverse vertebral diameter is ascribed to the increasing weight of the body. On the other hand, another study got a lower value of mean vertebral body transverse diameter at L5 than L4.^14^ Variation in vertebral morphometry is common in different parts of the world due to racial and ethnic variations.

The vertebral canal-body ratio showed a drop to 0.49 at L4 followed by an increase at L5. The mean canal-body ratio was observed to be 0.53. The exact same pattern has been reported in a study, where the canal body ratio gradually decreased from L1 to L4 and increased at the L5 level.^8^ However, the canal-body ratio was not constant at all levels. A study conducted in South Africa also showed the same pattern of the drop of ratio from L1 to L4 and relative increase in L5, where the parameters have been taken from the midsagittal dimensions.^15^ In the study, in dry bones, they recorded the canal-body ratio to be constant at both L1 and L2 levels which dropped at L3 with an increase at L4 and finally, the ratio again dropped at L5.^6^ Variations in the canal body ratio have been recorded in different kinds of literature. Literature has reported a constant canal body ratio,^1,16^ whereas on the other hand, it was also observed that the canal-body ratio was not found constant at any vertebral level.^17^ This variation can be due to genetic and environmental factors.

The AP of the canal was observed to be almost constant till L4 with a slight increase at L5. The mean AP diameter of the canal showed a gradual decrease from L1 to L4 vertebral level then increased at L5 level in the study.^7^ There are few data in the previous studies which showed a craniocaudal increase in the AP diameter of the canal.^13,18^ In contrast, some studies depicted a craniocaudal decrease in the AP diameter from L1 to L5.^8,19^

The mean AP diameter of the vertebral body in the present study showed a gradual increase with a minimum at L1 and a maximum at L5. This finding was similar to the study done.^7^ where the mean AP diameter was maximum at the L5 level following a gradual increase from L1 to L4 vertebral level.^7^ In contrast, one of the study found the gradual increment of vertebral body AP diameter from L1 to L3 and decreased at L4 and it was maximum at L5.^14^ The increase in the size of lower lumbar vertebrae can be attributed to it being the area for various movements and the weightbearing actions. Whereas for the variations, the racial, ethnic, and environmental factors could be the reasons coming into play.

Despite the different factors influencing the variations observed, the present study was on the limited available bones, the number and set of which could have been broadened. Also, the single centre nature of the study limits the generalizability of the findings. Further, as this was a descriptive cross-sectional study, the association between the variables could not be made.

## CONCLUSIONS

The mean canal-body ratio observed in the present study was similar when compared to other studies done in a similar setting. Different factors like ethnicity, racial and geographical distribution can have an influence on the vertebral body morphology. Nevertheless, the anatomical knowledge of the dimensions and canal-body ratio of the lumbar vertebrae may be helpful for clinicians for a better understanding of the lumbar spine pathology.
